# The Society of Community Medicine

**Published:** 1973-07

**Authors:** 


### The Society of Community Medicine

### West of England Branch

The West of England Branch of the Society of Medical Officers of Health has about 150 members and holds quarterly meetings, commonly at Post Graduate Medical Centres. Following a very successful course on Developmental Paediatrics in 1967, organised in co-operation with the Bristol University Department of Child Health and Public Health, the Branch was able to make substantial donations to the Parent Society and to the Jenner Trust and smaller contributions towards the funds of all the Post Graduate Medical Centres in the West of England.

In April 1973 the Society of Medical Officers' of Health changed its name to the Society of Community Medicine. In January 1973 the West of England Branch (President—Dr. D. K. MacTaggart, Torbay) held a meeting at Frenchay Postgraduate Centre, Frenchay Hospital, when Dr. John S. M. Zorab talked about "Resuscitation? a child could do it!" In April, Dr. D. C. Morgan, Head of the Epidemiology Division, Institute of Biometry and Community Medicine, University of Exeter, gave a talk entitled "Epidemiology and the evaluation of the treatment of Coronary Thrombosis" at the Torbay Postgraduate Centre. In June, at Bath, three members of the Branch, Dr. C. D. L. Lycett (Trowbridge) and Drs. P. N. Dixon and S. J. P. Woods (Bristol), talked about "Prospects and Training in Community Medicine".

The officers for the Branch for the 1973/4 session are:—

President: Dr. J. F. Skone.

President elect: Dr. D. O. McKnight (Exeter).

Hon. Secretary: Dr. Roy Barnes (Gloucester)

and representative on the Council of the Society: Dr. H. Binysh (Truro). The Deputy representative on the Council and Secretary of the Southern Area of the Branch is Dr. Mary Budding (Tavistock).

Dr. Skone will give his Presidential Address in Taunton on the 6th October. During the 1973/4 Session it is hoped to arrange joint meetings with other professional associations, including an all day meeting with the Mid-West Veterinary Association at the University of Bristol Veterinary School at Langford.

